# Beyond the *FTO* Gene: Environmental and Behavioural Factors Associated with BMI and Overweight in Spanish Adolescents

**DOI:** 10.3390/children13060782

**Published:** 2026-06-04

**Authors:** Luciana Margara, Inés Piñas-Bonilla, Pablo Abián, Alfredo Bravo-Sánchez, David Ortiz-Sánchez, María Ramírez-delaCruz, Paula Esteban-García, Javier Portillo, Carlos Ramírez, Javier Abián-Vicén

**Affiliations:** 1 Performance and Sport Rehabilitation Laboratory, Faculty of Sports Sciences, University of Castilla-La Mancha, 45071 Toledo, Spain; luchimargara452@gmail.com (L.M.); david.ortiz@uclm.es (D.O.-S.); maria.ramirez@uclm.es (M.R.-d.); paula.esteban@uclm.es (P.E.-G.); luis.portillo@uclm.es (J.P.); carlos.ramirezdelacr@uclm.es (C.R.); 2 Faculty of Medicine, University of Extremadura, 06006 Badajoz, Spain; inespibonilla@gmail.com; 3 Department of Biomedical Sciences, Area of Physical and Sports Education, Faculty of Medicine and Health Sciences, Universidad de Alcalá, 28871 Alcalá de Henares, Spain; pablo.abian@uah.es; 4 Faculty of Health Sciences, Universidad Francisco de Vitoria, 28223 Pozuelo de Alarcón, Spain; alfredo.bravo@ufv.es

**Keywords:** *FTO* gene, overweight, genetics, adolescents, physical activity

## Abstract

**Highlights:**

**What are the main findings?**

- The *FTO* rs9939609 polymorphism was not associated with BMI, weight status, or lifestyle-related variables in Spanish adolescents, suggesting a limited role of this genetic factor during this developmental stage.
- Environmental and behavioural factors, particularly regular breakfast consumption, showed a significant association with lower overweight prevalence, while notable sex differences were observed in physical fitness and activity levels.

**What are the implications of the main findings?**

- Preventive strategies targeting adolescent overweight should prioritize modifiable lifestyle behaviours, especially the promotion of regular breakfast consumption and increased physical activity.
- Public health interventions should incorporate sex-specific approaches, acknowledging the lower physical activity and fitness levels observed in girls, to enhance the effectiveness of obesity prevention programmes in adolescence.

**Abstract:**

**Background/objectives:** Obesity is a multifactorial condition influenced by interactions between genetic susceptibility and environmental factors. The fat mass and obesity-associated (*FTO*) gene has been widely linked to obesity risk, particularly the rs9939609 polymorphism, which is associated with higher body mass index (BMI) and adiposity. However, evidence in adolescents remains inconsistent, and lifestyle factors such as physical activity and diet may modify genetic risk. The objectives of this study were: (i) to examine the influence of environmental, genetic, physical activity, and dietary factors on the BMI and overweight-related variables of adolescents, and (ii) to assess the impact of the rs9939609 polymorphism in the *FTO* gene on these variables. **Methods:** A cross-sectional study was conducted involving 206 adolescents aged 12 to 16 years. Body mass index (BMI), physical fitness, physical activity levels, adherence to the Mediterranean diet, mobile phone usage, and *FTO* rs9939609 genotyping from buccal swabs were collected. **Results:** No significant associations were found between the *FTO* genotype and BMI, or with physical activity, mobile phone usage and dietary habits. Boys showed higher physical fitness and physical activity levels than girls (*p* < 0.05). The only factor significantly associated with BMI was regular breakfast consumption: adolescents who habitually ate breakfast had a lower prevalence of overweight (χ^2^ = 7.98, *p* = 0.005). **Conclusions:** The rs9939609 polymorphism in the *FTO* gene was not associated with overweight in this adolescent cohort. The findings underscore the relevance of healthy behaviours, particularly regular breakfast consumption and physical activity, especially among boys, as factors potentially associated with lower prevalence of overweight during adolescence.

## 1. Introduction

With the advancement of genome-wide association studies (GWAS), evidence has emerged highlighting the importance and involvement of the genetic component in the onset and development of obesity [1,2]. The genetic basis of obesity is commonly attributed to variations in multiple genes, and the fat mass and obesity-associated (*FTO*) gene has been widely described in the literature as being strongly associated with obesity. Among the various *FTO* gene variants, the rs9939609 (A/T) polymorphism in intron one of the *FTO* gene is one of the most significant genetic factors for susceptibility to obesity, as individuals carrying the A allele exhibit a higher body mass index (BMI) compared to those with the TT genotype in adulthood [3,4]. *FTO* is ubiquitously expressed in human tissues, with its highest levels found in the hypothalamic nuclei, which regulate energy balance [5]. Variants within the *FTO* gene have been linked not only to increased adiposity in humans, but also to the development of metabolic syndrome and cardiovascular disease [5,6].

Fat accumulation during childhood is associated with health complications that may persist into adulthood [7]. Physical inactivity and excessive energy intake are the main factors associated with the rising prevalence of obesity and overweight in children and adolescents, which are linked to lower levels of cardiorespiratory fitness and an increased presence of cardiovascular risk factors [5]. Although the relationship between the *FTO* gene and susceptibility to obesity has been consistently demonstrated, lifestyle-related factors may influence the obesity risk associated with this gene. Previous studies have indicated that the effect of *FTO* variants on body fat accumulation could be reduced among individuals who engage in regular physical activity [8,9].

Both modifiable and non-modifiable risk factors for the development of obesity have been identified [10]. Among the latter, several studies have reported a strong association between the rs9939609 polymorphism of the *FTO* gene and obesity in children and adolescents [11,12]. Among the modifiable risk factors, diet and physical activity are the two fundamental pillars determining body composition [13]. Several studies have shown that the Mediterranean diet not only acts as a protective factor against obesity, but also represents the most effective dietary model for weight loss [14,15]. Physical activity, meanwhile, has been profoundly impacted by the global rise in sedentary behaviour [16]. Moreover, it has been observed that individuals with a less favourable genetic predisposition require higher levels of exercise intensity to achieve benefits comparable to those with a more advantageous genetic profile [17,18]. This is further compounded by the progressive replacement of physical activity with sedentary behaviours based on the use of interactive devices [19], which may be particularly detrimental when coexisting with other obesity-related risk factors [20].

The progressive increase in overweight and obesity among children and adolescents worldwide remains a subject of ongoing research. Understanding how modifiable and non-modifiable risk factors interact is essential for the development of early prevention strategies [1]. Despite the well-established association between the *FTO* rs9939609 polymorphism and obesity risk, limited evidence is available regarding the combined influence of genetic predisposition, physical activity, dietary habits, and physical fitness on overweight-related outcomes in adolescents. In addition, studies simultaneously examining these interactions in Spanish adolescent populations and considering potential sex-related differences remain scarce. Therefore, the present study aimed to: (i) determine the influence of various factors related to the environment, genetics, physical activity, and dietary habits on BMI and overweight-related variables in adolescents, and (ii) assess the impact of the rs9939609 polymorphism of the *FTO* gene on the aforementioned factors.

## 2. Materials and Methods

### 2.1. Participants

A cross-sectional observational study was conducted on a sample of 206 students (boys: *n* = 119, age = 13.6 ± 1.0 years, body mass = 55.5 ± 12.5 kg, height = 163.6 ± 9.6 cm; girls: *n* = 87, age = 13.4 ± 0.9 years, body mass = 52.2 ± 8.7 kg, height = 159.4 ± 6.5 cm), aged between 12 and 16 years, enrolled in compulsory secondary education in state and state-subsidised schools in the province of Toledo, Spain. School selection was based on geographical and sociodemographic criteria to ensure diverse representation. Students who regularly attended school and appeared to be in good health were included. Exclusion criteria were as follows: students with chronic conditions such as diabetes mellitus, arterial hypertension, or other known chronic diseases were excluded from the study. In addition, students undergoing pharmacological treatment were not included, due to the potential influence on anthropometric, metabolic, or genetic outcomes.

The study was approved by the Ethics Committee of the General Hospital Complex in Toledo (Ref: 2304/2025), in accordance with all the principles outlined in the Declaration of Helsinki for research involving human subjects. In addition, informed consent was obtained from all parents or legal guardians, along with authorisation for participation from all the educational institutions involved and the regional government of Castilla-La Mancha.

### 2.2. Measurement Protocol

Researchers visited the schools on two separate occasions. On the first day, the objectives of the study and the tests to be conducted were explained to the participants, and questionnaires were completed during school hours in the classrooms under the supervision of the research team and the teachers. Students completed the International Physical Activity Questionnaire (IPAQ), the KIDMED questionnaire for assessing adherence to the Mediterranean diet in children and adolescents, and the Mobile-Related Experiences Questionnaire (CERM), all in validated Spanish versions previously used in school-aged populations. A saliva sample was collected using a buccal swab to extract genomic DNA and identify the rs9939609 polymorphism of the *FTO* gene. On the second day, anthropometric measurements were taken to calculate BMI, and the ALPHA-Fitness test battery was conducted alongside the 20-m shuttle run test (Course-Navette) to assess aerobic capacity. All participants completed the questionnaires, anthropometric assessments, genetic sampling, and physical fitness tests; therefore, no missing data were recorded for the variables included in the analyses. The following sections describe the protocols used for each of the tests and questionnaires:

#### 2.2.1. Body Mass Index

Anthropometric measurements were taken with each participant wearing light underwear and no shoes. Body mass and height were measured using a Seca electronic scale with stadiometer (Seca 769, Seca GMBH, Hamburg, Germany), with a precision of 0.1 kg for body mass and 0.1 cm for height. BMI was calculated as the ratio of body mass (kg) to height squared (m^2^). All anthropometric assessments followed the protocols established by the International Society for the Advancement of Kinanthropometry (ISAK) [21]. Each student’s percentile was determined based on their BMI value, taking into account age and sex, according to the reference tables provided by the World Health Organisation. Students were classified into BMI percentile categories as follows: <5% underweight, 5–85% normal weight, >85% overweight, and >95% obese [22]. BMI percentiles are widely used in epidemiological studies involving adolescents due to their practicality, accessibility, and established association with overweight and obesity risk [23].

#### 2.2.2. International Physical Activity Questionnaire (IPAQ)

The short version of the IPAQ (9 items) was used to assess physical activity related to aerobic capacity in adolescents. This version accounts for physical activity lasting at least 10 min, including leisure-time activities, household tasks, work-related activities, and active transportation over the past seven days. Physical activity levels are classified based on weekly energy expenditure, expressed in metabolic equivalent tasks (MET) minutes/week, where one MET represents resting energy expenditure. A MET score is calculated for walking, moderate activity, and vigorous activity (3.3, 4.0, and 8.0 MET min/week, respectively), and a total index is obtained by summing the MET min/week for each intensity level of physical activity [24]. Numerous review studies support the IPAQ’s high level of validity [25,26].

#### 2.2.3. Questionnaire for Assessing Adherence to the Mediterranean Diet in Children and Adolescents (KIDMED)

The KIDMED questionnaire (Mediterranean Diet Quality Index) is a tool used to evaluate adherence to the Mediterranean diet, nutritional status, and overall diet quality in children and adolescents [15]. Participants completed a seven-day food diary, covering both weekdays and the full weekend. The assessment included a 16-item Mediterranean Diet Quality Index, a 169-item quantitative food frequency questionnaire, and a general questionnaire addressing socioeconomic, demographic, and lifestyle factors. Scores were categorised into three levels of adherence to the Mediterranean diet: low (≤1), moderate (2–4), and high (≥5). In addition, one specific item from the KIDMED questionnaire—whether or not adolescents ate breakfast—was analysed separately, as several studies have identified an association between breakfast skipping and an increased risk of overweight and obesity in adolescents [27,28]. Upon completion of data collection, the information was analysed using a nutrition software programme specifically designed for the Mediterranean diet by the Centre for Higher Education in Nutrition and Dietetics (CESNID; PCN Cesnid 1.0, Barcelona, Spain).

#### 2.2.4. Mobile-Related Experiences Questionnaire (CERM)

The CERM is a questionnaire addressing experiences related to mobile phone use. It consists of 10 items rated on a four-point Likert scale, where 1 corresponds to “almost never” and 4 to “almost always” [29]. The questionnaire demonstrates good overall reliability and evaluates two factors: the presence of conflicts, and the communicational and emotional use of mobile phones and social networks among young people [30].

#### 2.2.5. Physical Fitness (ALPHA-Fitness)

Several tests from the ALPHA-Fitness battery were conducted; this is a standardised set of physical assessments designed to measure health-related physical fitness in children and adolescents aged 6 to 18 years [31]. The tests performed in our study were as follows:

- Handgrip Strength: Measured using a manual dynamometer (TKK 5401 Grip D, Takei, Tokyo, Japan). During the test, students were instructed to squeeze the dynamometer slowly and steadily for 3 to 5 s. Two trials were performed alternately with both hands, recording the highest value obtained for each hand.
- Medicine Ball Throw: Participants threw a 2 kg medicine ball overhead as far as possible using trunk and upper limb extension and flexion. Two attempts were made per student, with the distance of each throw recorded; the longest distance was used for analysis.
- Standing Long Jump: The student stood behind a marked line on the floor with feet together and performed a maximal jump, landing evenly on both feet. Any unbalanced landing was considered a null attempt. Each student performed two attempts, with the distance measured in centimetres using a tape measure; the longest jump was used for subsequent analysis.
- Cardiorespiratory Capacity: Assessed via the Course-Navette test (20-m shuttle run). Participants ran back and forth over a 20-m course, keeping pace with audio beeps starting at 8.5 km/h and increasing by 0.5 km/h each minute. The test ended when a participant stopped due to fatigue or failed to reach the line before the beep. The time recorded for each participant was noted.

#### 2.2.6. Genetics

Genomic DNA was obtained from buccal epithelial cells using a cotton swab according to a previously described protocol [32], and genotyping was performed in a certified genetics laboratory. To minimize the risk of contamination, standard recommendations for molecular genetics laboratories were strictly applied, including the use of separate physically isolated areas for each procedure, such as sample handling and DNA extraction. Following collection, the samples were stored at 4 °C and subsequently transported to the laboratory. Upon arrival, genomic DNA extraction was carried out automatically using the QIACube system (QIAGEN, Venlo, The Netherlands) to achieve a final DNA concentration of at least 25 ng/mL. The resulting solution was subsequently stored at −20 °C until genotyping analysis, which was carried out within one week after sample receipt at the laboratory. During genotyping, the rs9939609 polymorphism of the *FTO* gene (Fat Mass and Obesity-Associated protein), linked to the likelihood of excess adiposity, was analysed. All samples for which genotype determination was inconclusive were reanalyzed. In addition, reference samples, including internal controls, blank samples, and negative controls, together with continuous contamination monitoring, were incorporated throughout every stage of the procedure. Results were analysed using the 7500 v 2.0.5 software (Applied Biosystems, Foster City, CA, USA). The genotyping success rate for this sample was 100%.

### 2.3. Statistical Analysis

Statistical analyses were conducted using IBM SPSS Statistics version 28.0 (SPSS Inc., Chicago, IL, USA). Categorical variables are reported as absolute frequencies and percentages, whereas continuous variables are expressed as mean ± standard deviation. The distribution normality of continuous variables was evaluated using the Kolmogorov–Smirnov test, with all variables showing a parametric distribution (*p* > 0.05). The Chi-square test (χ^2^) was used to verify that genotype frequencies conformed to the Hardy–Weinberg equilibrium (HWE). The χ^2^ test was also applied to determine whether the genotype frequency in the study sample differed from that recorded in ethnically matched controls from the “1000 Genomes Database” [33], and to examine the association between breakfast habits and groups defined by BMI percentiles (normal weight, overweight, and obesity). A two-way ANOVA (3 × 2) was conducted to determine the main effects of genotype and sex on the physical variables and questionnaire outcomes analysed. The ANOVA factors were genotype (AA, AT, and TT) and sex (boys vs. girls). When significant main effects were detected in the ANOVA, the Bonferroni correction was applied as a post-hoc test. In addition, pairwise comparisons were complemented with the calculation of 95% confidence intervals (95% CI) for the mean differences. Effect sizes were calculated using Cohen’s d for pairwise comparisons and Cramér’s V for categorical associations (breakfast consumption and BMI categories). Associations between variables were evaluated using Pearson’s correlation coefficient for continuous variables, whereas Spearman’s rank correlation coefficient was applied when categorical variables were involved. A *p*-value < 0.05 was considered indicative of statistical significance in all analyses.

## 3. Results

Table 1 shows the frequency distribution of the different genotypes of the *FTO* polymorphism in the schoolchildren. The distribution of *FTO* gene genotypes met the Hardy–Weinberg equilibrium (χ^2^ = 3.44, *p* = 0.064). The distribution of the AA, AT and TT genotypes was similar between the boys’ and girls’ groups (χ^2^ = 0.94, *p* = 0.627). Furthermore, the genotype frequencies observed in the sample of schoolchildren were comparable to those reported in the 1000 Genomes Database (χ^2^ = 1.82, *p* = 0.403).

Table 2 shows that no association was found (χ^2^ = 1.48, *p* = 0.829) between the *FTO* gene genotype (AA, AT and TT) and the groups generated based on BMI percentiles adjusted for sex and age (normal weight: between the 5th and 85th percentile; overweight: between the 85th and 95th percentile; obesity: above the 95th percentile).

No significant interaction (sex × group) was found for any of the variables related to physical fitness assessments (Table 3). A significant main effect of sex (*p* < 0.05) was observed across all physical performance variables. Compared to the girls’ group, the boys showed a better performance in all tests analysed: standing long jump performance was 13.3% longer (difference = 0.21 m; 95% CI [0.07, 0.34]; F = 2.99; *p* = 0.003; Cohen’s *d* = 0.72); medicine ball throw distance increased by 10.9% (difference = 0.52 m; 95% CI [0.03, 1.01]; F = 2.09; *p* = 0.039; Cohen’s *d* = 0.48); right-hand grip strength was 21.4% higher (difference = 5.31 kg; 95% CI [3.02, 7.61]; F = 4.56; *p* < 0.001; Cohen’s *d* = 0.81), while left-hand grip strength showed a 19.0% difference (difference = 4.27 kg; 95% CI [2.30, 6.24]; F = 4.28; *p* < 0.001; Cohen’s *d* = 0.76). Similarly, in the Course-Navette cardiovascular endurance test, boys recorded a 43.3% higher performance than girls (difference = 2.19 min; 95% CI [1.32, 3.06]; F = 4.98; *p* < 0.001; Cohen’s *d* = 1.19) (Table 3).

No significant interaction (sex × group) was found, nor any main effect of genotype or sex on BMI, IPAQ, CERM, or KIDMED scores, except for the IPAQ, where a significant main effect of sex was observed. Boys scored 50.4% higher than girls (difference = 2092 points; 95% CI [1030, 3154]; F = 3.88; *p* < 0.001; Cohen’s *d* = 0.69). No significant correlations were found between BMI and the other variables analysed in Table 4: IPAQ (r = 0.01; *p* = 0.934), CERM (r = −0.06; *p* = 0.403), KIDMED (r = 0.086; *p* = 0.219), and Genotype (ρ = −0.04; *p* = 0.615).

A significant association was found (χ^2^ = 7.98, *p* = 0.005, Cramer’s V = 0.20) between students who regularly ate breakfast and the groups defined according to BMI. A higher percentage of students who regularly consumed breakfast was observed in the normal weight group (5th–85th percentile) compared to the overweight and obesity group (percentile > 85%) (Table 5).

## 4. Discussion

The aim of this research was to determine the influence of various factors, such as environment, genetics, physical activity, and nutritional habits, on adolescents’ BMI and overweight, in order to identify tools that may help predict modifiable risk factors and implement timely prevention strategies. In this study, no associations were found between modifiable and non-modifiable factors and BMI. Notably, no relationship was observed between the *FTO* gene genotype (AA, AT, and TT) and the BMI-based groups (normal weight, overweight, and obesity) defined by age- and sex-specific percentiles. This suggests that carrying the risk allele of the *FTO* gene at the ages studied (12–16 years) does not necessarily predispose individuals to being classified in the overweight or obese categories. However, these findings should be interpreted cautiously, as the limited number of participants with obesity may have reduced the statistical power to detect genotype-related differences. On the other hand, boys performed better in the physical fitness tests and reported higher levels of daily physical activity, as measured by the IPAQ questionnaire, compared to girls. Regarding eating habits, regular breakfast consumption was associated with lower prevalence of overweight among adolescents.

The distribution of *FTO* genotypes in this study conformed to the Hardy–Weinberg equilibrium. Furthermore, the distribution of the AA, AT, and TT genotypes was similar between boys and girls. Likewise, no differences were found in the distribution of *FTO* genotypes (AA, AT, and TT) across the groups classified by BMI percentiles according to age and sex. In contrast to our findings, most authors report that individuals carrying the A allele exhibit higher BMI levels compared to those with the TT genotype in adulthood [2]. However, in agreement with our results, some studies have found no association between the *FTO* gene and BMI [34]. In the present study, no significant association was observed between the rs9939609 polymorphism and BMI categories. This finding should be interpreted cautiously considering the characteristics of the sample, particularly the predominance of adolescents with normal BMI values and the exploratory nature of the study. Furthermore, the limited statistical power of the genetic analyses may have reduced the ability to detect small genotype-related effects. Consequently, larger studies are required before firm conclusions can be drawn regarding the absence of an association between the rs9939609 polymorphism and obesity-related outcomes in adolescents. Nevertheless, previous research has suggested that the integration of genetic information with lifestyle-related factors may contribute to the individualisation of obesity prevention strategies in adolescents [35]. Additionally, in terms of participant age, some studies suggest that many genes associated with development may not be expressed until adulthood [36], and that the influence of factors such as physical activity, social environment, and nutritional habits is less significant in children and adolescents than in adults [37].

Boys demonstrated a higher performance than girls in all physical fitness tests. These differences are accentuated as a result of pubertal development, which involves sex-specific hormonal and morphological changes. Such differences have been identified in physical variables similar to those assessed in our study from the age of 12 onwards, across various adolescent populations [38,39]. Pate et al. [40], in a study involving 4732 American adolescents aged between 12 and 19 years, found that boys’ cardiorespiratory fitness was 11% higher than that of girls at age 12, with this difference increasing to 21% by age 18. Furthermore, in our study, boys reported 50.4% higher weekly physical activity levels than girls. As noted by Armstrong and Welsman [41], sex differences in physical activity levels have been identified in several studies conducted across different European countries. These differences are attributed to varying interests, abilities, and preferences between boys and girls, as well as to social and logistical barriers that hinder equitable participation, particularly in higher-intensity activities, where boys tend to report higher values [42,43]. Given the lower levels of physical activity observed among girls, future prevention programmes should consider sex-specific approaches designed to reduce social and environmental barriers and promote greater participation in physical activity among adolescent females.

No patterns of problematic mobile phone use were identified among the adolescents assessed, nor were any differences observed in adherence to the Mediterranean diet or in physical activity levels according to *FTO* genotype. These findings are consistent with previous research in which no associations were found between the *FTO* gene, specifically the rs9939609 variant, and either energy intake or physical activity levels in children and adolescents [44,45]. The *FTO* gene is expressed in various tissues, including those involved in appetite regulation within the central nervous system and adipose tissue [46], Previous studies have suggested a potential role of *FTO* in lipolysis, although its precise function remains unclear [47]. Its high expression in the hypothalamus suggests an involvement in the regulation of appetite and energy homeostasis [48]. However, as in our study, the rs9939609 polymorphism has not been associated with energy expenditure or physical activity levels in prior investigations [44,45]. On the other hand, although our study did not find an association with adherence to the Mediterranean diet, some research indicates that this polymorphism may influence appetite and food choices, reflected in higher energy and fat intake [49].

Regarding dietary habits, we found that adolescents who regularly consumed breakfast had lower BMI values than those who did not. While some studies have reported no consistent association between breakfast consumption and BMI or the prevalence of overweight/obesity [39,50], several investigations indicate that regular breakfast intake is associated with lower body fat and a reduced risk of obesity [51,52]. The evidence supports a correlation between regular breakfast consumption and lower obesity rates; however, it is important to acknowledge the complexity of eating behaviours and their multifactorial influence on obesity. In line with our findings, a study conducted among Malaysian adolescents reported that those who consumed breakfast at least five times per week had significantly lower body weight, BMI, waist circumference, and body fat percentage compared to those who ate breakfast less frequently [53]. These findings support the implementation of school-based interventions aimed at promoting healthy breakfast habits as part of broader obesity prevention strategies during adolescence. Nevertheless, this association should be interpreted with caution given the cross-sectional design of the study and the large number of statistical comparisons performed. As such, the finding should be considered exploratory and confirmed in future longitudinal studies.

This study presents certain limitations that help contextualise the scope of its findings. Firstly, although the sample size is adequate for most of the analyses performed, it is limited for genetic studies, which typically require larger cohorts due to the small effect sizes associated with specific variants. This study should also be interpreted considering that no a priori statistical power calculation was performed for the genetic analyses. In particular, the small number of participants with obesity may have reduced the statistical power to detect genotype-related differences. Nevertheless, the sample size was comparable to that of previous exploratory studies conducted in adolescent populations. It is important to recognise that carrying a particular *FTO* genotype is not the sole factor contributing to the development of overweight or obesity, as other genes and epigenetic modifications may predispose adolescents to higher-than-normal BMI. In addition, biological maturation and pubertal status were not assessed, which may represent a potential confounding factor influencing BMI and physical fitness outcomes during adolescence. With regard to body composition assessment, BMI was used; although it is a widely accepted and accessible tool, it does not allow for an accurate analysis of body fat composition in this study. Finally, it should be noted that the dietary assessment methods and the self-administered questionnaires used to estimate physical activity levels have inherent limitations in terms of accuracy. Although the IPAQ has been previously used and validated in adolescent populations, it was originally developed for adults, which may limit the precision of physical activity assessment in participants aged 12–16 years. Self-reported questionnaires may also be affected by recall bias and social desirability bias, potentially influencing the observed associations. Additionally, the large number of statistical comparisons performed may increase the risk of type I error.

## 5. Conclusions

The findings of the present study do not reveal significant associations between BMI and either modifiable factors (dietary habits, adherence to the Mediterranean diet, physical activity level, physical performance) or non-modifiable factors (rs9939609 *FTO* gene genotype). Specifically, the presence of the *FTO* risk allele was not associated with overweight or obesity status among adolescents in this sample. However, these findings should be interpreted with caution, as the small number of participants with obesity may have limited the statistical power of the genetic analyses. Sex-based differences were identified, with boys demonstrating better physical performance and higher levels of daily physical activity. Furthermore, adolescents with normal BMI values reported regular breakfast consumption more frequently than those with overweight or obesity, although the cross-sectional design of the study does not allow causal relationships to be established.

Although no direct correlations were found between the evaluated factors and BMI, the results support the development of preventive strategies, highlighting the importance of fostering environments that do not promote overweight and obesity. The interaction between genetic, environmental, physical activity, and nutritional factors underscores the need for a multidimensional approach to understanding and preventing overweight and obesity during adolescence.

## Figures and Tables

**Table 1:** Number (frequency) of schoolchildren according to their *FTO* gene genotype (AA, AT and TT) and allele distribution (A and T), compared with ethnically matched controls from the 1000 Genomes Database [33] (only frequency, in %).

Group	Genotype Frequency			Allele Frequency	
	AA	AT	TT	A	T
All (*n* = 206)	24 (11.7)	110 (53.3)	72 (35.0)	158 (38.3)	254 (61.7)
Boys (*n* = 119)	16 (13.4)	63 (52.9)	40 (33.6)	95 (39.9)	143 (60.1)
Girls (*n* = 87)	8 (9.2)	47 (54.0)	32 (36.8)	63 (36.2)	111 (63.8)
1000 genome database; European	16.8	48.4	34.8	41.0	59.0

**Table 2:** Association between *FTO* gene genotype and groups classified by body mass index (BMI) percentile. [number (frequency)].

Group	Genotype Frequency			
	AA	AT	TT	Total
Normal	19 (11.5)	87 (52.7)	59 (35.8)	165 (100.0)
Overweight	3 (9.7)	17 (54.8)	11 (35.5)	31 (100.0)
Obesity	2 (20.0)	6 (60.0)	2 (20.0)	10 (100.0)
All	24 (11.7)	110 (53.4)	72 (35.0)	206 (100.0)

**Table 3:** Physical fitness assessments (mean ± SD) from the ALPHA-Fitness battery stratified by *FTO* rs9939609 genotype (AA, AT, TT) and by sex in the sample of 206 schoolchildren. Results of two-way ANOVA (sex × genotype) are included.

	AA	AT	TT	Sex × Genotype	Sex	Genotype
Standing long jump (m)						
Boys	1.65 ± 0.33	1.72 ± 0.31	1.68 ± 0.31	0.832	0.003	0.463
Girls	1.38 ± 0.17	1.54 ± 0.28	1.51 ± 0.14			
Medicine ball throw (m)						
Boys	4.81 ± 1.07	5.16 ± 1.01	5.11 ± 1.30	0.140	0.039	0.529
Girls	5.12 ± 2.06	4.34 ± 0.82	4.06 ± 1.04			
Handgrip strength right (kg)						
Boys	25.9 ± 7.8	29.2 ± 8.3	27.3 ± 7.6	0.899	<0.001	0.110
Girls	20.7 ± 2.7	23.4 ± 3.8	22.4 ± 4.3			
Handgrip strength left (kg)						
Boys	22.9 ± 5.8	26.7 ± 7.1	24.3 ± 6.5	0.591	<0.001	0.081
Girls	19.3 ± 3.6	21.3 ± 3.9	20.5 ± 3.4			
Course navette (min)						
Boys	6.60 ± 1.39	6.20 ± 2.01	5.66 ± 2.26	0.708	<0.001	0.693
Girls	3.90 ± 2.56	4.04 ± 1.41	3.95 ± 1.08			

**Table 4:** Mean ± SD scores for Body Mass Index (BMI), the International Physical Activity Questionnaire (IPAQ), the Mobile-Related Experiences Questionnaire (CERM), and the Mediterranean Diet Quality Index for children and adolescents (KIDMED), stratified by *FTO* rs9939609 genotype (AA, AT, TT) and by sex in the sample of 206 schoolchildren.

	AA	AT	TT	Sex × Genotype	Sex	Genotype
BMI (Kg/m^2^)						
Boys	20.9 ± 2.7	20.5 ± 3.6	20.5 ± 3.5	0.872	0.998	0.753
Girls	21.0 ± 3.7	20.7 ± 3.3	20.2 ± 2.5			
IPAQ (score)						
Boys	5236 ± 2848	4971 ± 3343	5387 ± 4053	0.777	<0.001	0.982
Girls	3079 ± 2037	3239 ± 2169	3001 ± 2201			
CERM (score)						
Boys	15.6 ± 2.9	15.9 ± 3.6	16.4 ± 3.1	0.770	0.121	0.635
Girls	16.3 ± 4.7	17.5 ± 4.4	17.3 ± 4.8			
KIDMED (score)						
Boys	5.63 ± 2.31	5.56 ± 2.40	5.03 ± 2.31	0.253	0.453	0.752
Girls	5.38 ± 4.03	4.55 ± 2.75	5.28 ± 2.13			

**Table 5:** Association between breakfast consumption and groups defined by body mass index (BMI) percentiles.

BMI	Breakfast		
	No	Yes	Total
Normal	43 (26.1)	122 (73.9)	165 (100.0)
Overweight and Obesity	20 (48.8)	21 (51.2)	41 (100.0)
All	63 (30.6)	143 (69.4)	206 (100.0)

## Data Availability

The data presented in this study are available in Zenodo at https://doi.org/10.5281/zenodo.18213638, upon reasonable request.

## Abbreviations

The following abbreviations are used in this manuscript:

BMI	Body mass index
GWAS	Advancement of genome-wide association studies
*FTO*	Fat mass and obesity-associated gene
IPAQ	International Physical Activity Questionnaire
KIDMED	Questionnaire for Assessing Adherence to the Mediterranean Diet in Children and Adolescents
CERM	Mobile-Related Experiences Questionnaire
ANOVA	Analysis of Variance
